# Outcomes and comparations of pediatric surgery about choledochal cyst with robot-assisted procedures, laparoscopic procedures, and open procedures: A meta-analysis

**DOI:** 10.3389/fped.2022.968960

**Published:** 2022-08-11

**Authors:** Siqi Xie, Yanbing Huang, Yuanbin He, Mingkun Liu, Dianming Wu, Yifan Fang

**Affiliations:** Department of Pediatric Surgery, College of Clinical Medicine for Obstetrics and Gynecology and Pediatrics, Fujian Children's Hospital, Fujian Medical University, Fuzhou, China

**Keywords:** robotic-assisted operation, meta-analysis, choledochal cyst (CC), pediatric surgery, laparoscope

## Abstract

**Background:**

Choledochal cysts (CC) are rare disorders characterized by congenital biliary dilatation of the intrahepatic or extrahepatic bile ducts and always relate to pancreaticobiliary maljunction. Robot-assisted surgery has been able to complete almost all pediatric endoscopic surgery nowadays. But evidence of the post-operative outcomes of robotic-assisted operation is limited, comparing with the laparoscopic operation and traditional open operation. The aim of this meta-analysis was to identify the advantages and deficiencies about robotic-assisted operation for CC.

**Methods:**

A meta-analysis of retrospective studies published in PUBMED, MEDLINE, Web of Science and China National Knowledge Infrastructure (CNKI). No date limit was used, with the last search on April 30, 2022. No publication restrictions or study design filters were applied.

**Results:**

Nine retrospective cohort studies with 1,395 patients [366 in the robotic-assisted operation group (RG), 532 in the laparoscopic operation group (LG) and 497 in the open operation group (OG)] were enrolled in our study. Subgroup analysis demonstrated the RG had significant longer operative time [standardized mean difference (SMD) = 1.59, 95% CI = (0.02, 3.16), *P* < 0.05], less blood loss [SMD = −1.52, 95% CI = (−2.71, −0.32), *P* < 0.05], shorter enteral feeding time [SMD = −0.83, 95% CI = (−1.22, −0.44), *P* < 0.001], shorter time to stay in the hospital [SMD = −0.81, 95% CI = (−1.23, −0.38), *P* < 0.001], fewer post-operative complications [Relative risk (RR) =1.09, 95% CI = (1.04, 1.13), *P* < 0.001] but higher expenses [SMD = 8.58, 95% CI = (5.27, 11.89), *P* < 0.001] than LG. While a significant older age [SMD = 0.46, 95% CI = (0.26, 0.66), *P* < 0.001], longer operative time [SMD = 3.96, 95% CI = (2.38, 5.55), *P* < 0.001] and shorter time to stay in the hospital [SMD = −0.93, 95% CI = (−1.62, −0.25), *P* < 0.05] than OG.

**Conclusions:**

Laparoscopic and robotic-assisted procedure are both safe and minimal invasive operational strategies. Robotic-assisted procedure may slowly surpass and has a trend to replace laparoscopy for its advantages. More experiences in robotic-assisted operation should be accumulated for the unexpected complexities, so as to be more stable in the younger age of children.

## Introduction

Choledochal cysts (CC) are rare disorders characterized by congenital biliary dilatation of the intrahepatic or extrahepatic bile ducts and always relate to pancreaticobiliary maljunction. The primary treatment for CC is entire cyst excision with Roux-en-Y hepaticojejunostomy, which was performed decades ago as a traditional open surgery in Japan (1). Minimally invasive surgery has become mainstream due to the advantages of cosmetic outlook and quick recovery after operative, the first laparoscopic CC resection with Roux-en-Y hepaticojejunostomy was reported in 1995 (2). Eleven years later, the first robotic laparoscope-assisted type I CC resection for a five-year-old girl was reported by Woo et al. (3). Subsequently, some reports on robotic-assisted operation about CC resection in children had been reported (4–12). However, the evidence of the post-operative outcomes of robotic-assisted operation is limited, comparing with the laparoscopic operation and traditional open operation. The aim of this meta-analysis was to identify the advantages and deficiencies about robotic-assisted operation for choledochal cysts in children.

## Methods

Reporting followed the Preferred Reporting Items for Systematic Reviews and Meta-Analysis (PRISMA) guidelines (13). We conducted a meta-analysis of retrospective studies published in PUBMED, MEDLINE, Web of Science and China National Knowledge Infrastructure (CNKI). No date limit was used, with the last search on April 30, 2022. No publication restrictions or study design filters were applied. The search strategy for PUBMED was as follows: [robot (all fields)] AND [pediatric (all fields)] AND [choledochal cyst (all fields)]. Reference lists from related articles were also scanned to broaden the search. A hand search was performed in all four databases.

Inclusion criteria were (1) the study reported the pediatric operation comparing the robotic-assisted operation group (RG), laparoscopic operation group (LG) and/or open operation group (OG) about cyst resection and Roux-en-Y hepaticojejunostomy; (2) the study reported at least one of those outcomes: operative time, blood loss, post-operative complications, length of hospital stay, and costs; (3) the study provided appropriate statistical estimates or counts; (4) the study was reported in English or Chinese only.

Exclusion criteria were (1) case reports (<5 cases); (2) review articles; (3) the study included only one surgical method; (4) conference abstracts; (5) no comparative outcomes in the study.

The following information: name of first author, year of publication, type of study, mean age, gender, number of populations, and primary outcomes, including operative time, blood loss, time to enteral feeding, hospital stays, post-operative complications and expenses were extracted. The Newcastle—Ottawa scale (NOS) score (14) for those cohort studies focuses on three categories: selection, comparability and outcome. The maximum stars of NOS score are nine stars. An article assessed ≥6 stars was considered to be of high quality and adopted in our study.

Statistical analysis was conducted by STATA version 12.0. Relative risk (RR) was applied for dichotomous variables, and standardized mean difference (SMD) was applied for continuous variables. Some study outcomes were reported as medians with ranges or mid-quartiles with ranges. According to the methods introduced by Luo et al. (15) and Wan et al. (16), those data were converted to means with deviations, thus the results for each group are presented as the mean ± standard deviation ($\bar{\text{x}}$ ± s). The *I*^2^ statistic was used to test the degrees of heterogeneity, the *P*-value of *I*^2^ < 0.05 was used to indicate high heterogeneity and vice versa. The random-effects model was applied to pool the high heterogeneity results and the fixed-effects model was used for low heterogeneity (*P*-value of *I*^2^ > 0.05; **Table 3**). Begg's Test and Egger's Test were performed to assess the risk of bias (**Table 4**), while Begg's funnel plots were applied. *P* < 0.05 was considered to be statistically significant in the text.

## Results

We identified 166 papers through the article search. After removing duplications, 31 records were excluded after title and abstract evaluation, and 126 records were excluded after full-text review because they did not meet the inclusion criteria. Finally, nine retrospective cohort studies with 1,395 patients [366 in the robotic-assisted operation group (RG), 532 in the laparoscopic operation group (LG) and 497 in the open operation group (OG)] were enrolled in our study.

### Characteristics of included studies

The baseline characteristics of the nine records were listed in Table 1. Table 2 exhibited the information of operations associated, such as operative time, blood loss, time to enteral feeding, hospital stays, post-operative complications, and expenses. The NOS scores were ranged from 6 to 8 stars, reflecting the quality of cohort studies. Gender and age at surgery were comparable across groups.

**Table 1:** Baseline characteristics of nine records included in the meta-analysis.

**References**	**Study type**		**Number of patients**	**Gender(M/F)**	**Age at surgery** **(months, years)**	**NOS** **scores**
Chi et al. (4)	R	RG LG	70 70	22/48 22/48	34.00 ± 27.71 m 36.21 ± 32.80 m	8
Chi et al. (6)	R	RG LG	85 85	28/57 28/57	33.0 m (21.5 ~ 67.0) 33.0 1m (19.0 ~ 63.5)	8
Lin et al. (5)	R	RG LG	24 27	9/15 11/16	30.13 ± 13.88 m (3 ~ 54) 33.56 ± 11.56 m (8 ~ 60)	6
Koga et al. (7)	R	RG LG	10 27	NR NR	5.6 ± 3.4 y (1.8–11.2) 5.2 ± 3.8 y (0.7–13.8)	6
Xie et al. (8)	R	RG LG OG	41 104 226	10/31 25/79 52/174	48.00 m (30.50–77.50) 28.00 m (8.75–53.00) 33.50 m (17.75–60.00)	8
Kim et al. (9)	R	RG OG	36 42	6/30 15/27	57.5 ± 55.6 m 30.2 ± 36.1 m	7
Xie et al. (10)	R	RG LG OG	54 118 229	12/42 29/89 53/176	46.00 m (29–76) 28.00 m (8.75–53.00) 34.00 m (17.5–60)	8
Dong et al. (11)	R	RG LG	21 82	7/14 24/58	3.85 ± 0.79 y 3.71 ± 0.67 y	6
Cai et al. (12)	R	RG LG	25 19	10/15 2/17	52.2 ± 47.5 m 26.9 ± 23.2 m	6

R, retrospective; LG, laparoscopic operation group; RG, robotic-assisted operation group; OG, open operation group; NR, not reported; M, Male; F, Female; NOS, Newcastle-Ottawa Quality Assessment Scale.

**Table 2:** Surgical details, complications, and post-operative outcomes of nine records enrolled in the meta-analysis.

**References**	**Study type**		**Operative time (minutes)**	**Blood loss**	**Time to enteral feeding (days)**	**Hospital stays (days)**	**Post-operative complications**	**Expense (¥)**
Chi et al. (4)	R	RG LG	229.50 (198.00–251.00) 172.00 (157.25–186.75)	6.81 ± 2.00 ml 23.24 ± 4.93 ml	3.71 ± 0.71 4.3 ± 0.75	6.94 ± 1.21 7.91 ± 1.47	1 7	NR NR
Chi et al. (6)	R	RG LG	272.3 ± 39.5 194.8 ± 22.5	7.0 ± 1.9 ml 30.2 ± 7.5 ml	3.4 ± 0.5 4.3 ± 0.7	6.4 ± 1.2 8.3 ± 1.7	4 12	NR NR
Lin et al. (5)	R	RG LG	217.63 ± 5.90 (207 ~ 233) 199.37 ± 5.13 (189 ~ 210)	7.04 ± 1.16 ml (6 ~ 10) 29.04 ± 18.21 ml (15 ~ 100)	2.19 ± 0.32 (2 ~ 2.5) 3.26 ± 0.75 (2 ~ 5)	5.21 ± 0.29 (5 ~ 6) 7.26 ± 4.13 (5 ~ 20)	1 12	NR NR
Koga et al. (7)	R	RG LG	654 ± 144^a^ 618 ± 96	0.7 ± 0.32 ml/kg 0.91 ± 0.5 ml/kg	5.4 ± 1.4 8.0 ± 2.3	7.4 ± 1.0 11.0 ± 2.4	0 1	NR NR
Xie et al. (8)	R	RG LG OG	180.61 ± 14.07 212.79 ± 34.94 115.88 ± 13.5	21.34 ± 9.42 ml 21.73 ± 11.44 ml 40.12 ± 55.51 ml	3.74 ± 0.16 3.86 ± 0.34 4.78 ± 0.43	7.5 ± 1.00 7.56 ± 1.08 10.28 ± 2.23	2 9 7	62,320 ± 3,798 35,430 ± 1,847 28,460 ± 2,615
Kim et al. (9)	R	RG OG	520 ± 97 327 ± 73	79 ± 183 ml 33 ± 52 ml	4 (3–22) 5 (4–6)	9.2 ± 4.0 9.7 ± 3.5	5 1	NR NR
Xie et al. (10)	R	RG LG OG	181.28 ± 14.07 216.14 ± 35.57 115.88 ± 13.41	21.85 ± 9.82 ml 21.82 ± 11.15 ml 40.12 ± 55.17 ml	2.97 ± 0.3 3.08 ± 0.37 4.07 ± 0.38	7.46 ± 0.92 7.54 ± 1.08 10.27 ± 2.34	2 9 7	62,320 ± 3,798 35,030 ± 2,047 28,450 ± 2,515
Dong et al. (11)	R	RG LG	290.13 ± 41.04 193.21 ± 26.73	7.82 ± 2.61 ml 11.33 ± 4.50 ml	3.82 ± 0.73 4.59 ± 1.27	7.40 ± 1.15 9.71 ± 1.83	1 5	NR NR
Cai et al. (12)	R	RG LG	189.4 ± 35.5 167.1 ± 33.9	5 (5–10) ml 10 (5–20) ml	3.5 (3–4) 4 (3–4)	8.7 ± 2.3 11.0 ± 2.5	2 1	75,000 (73,000–86,000) 32,000 (27,000–36,000)

R, retrospective; LG, laparoscopic operation group; RG, robotic-assisted operation group; OG, open operation group; NR, not reported; ¥, (1¥ = 0.149$).

a Include robot docking time.

### Comparations and outcomes of the meta-analysis

#### Age at surgery

##### RG v. sLG

**Figure 1:**
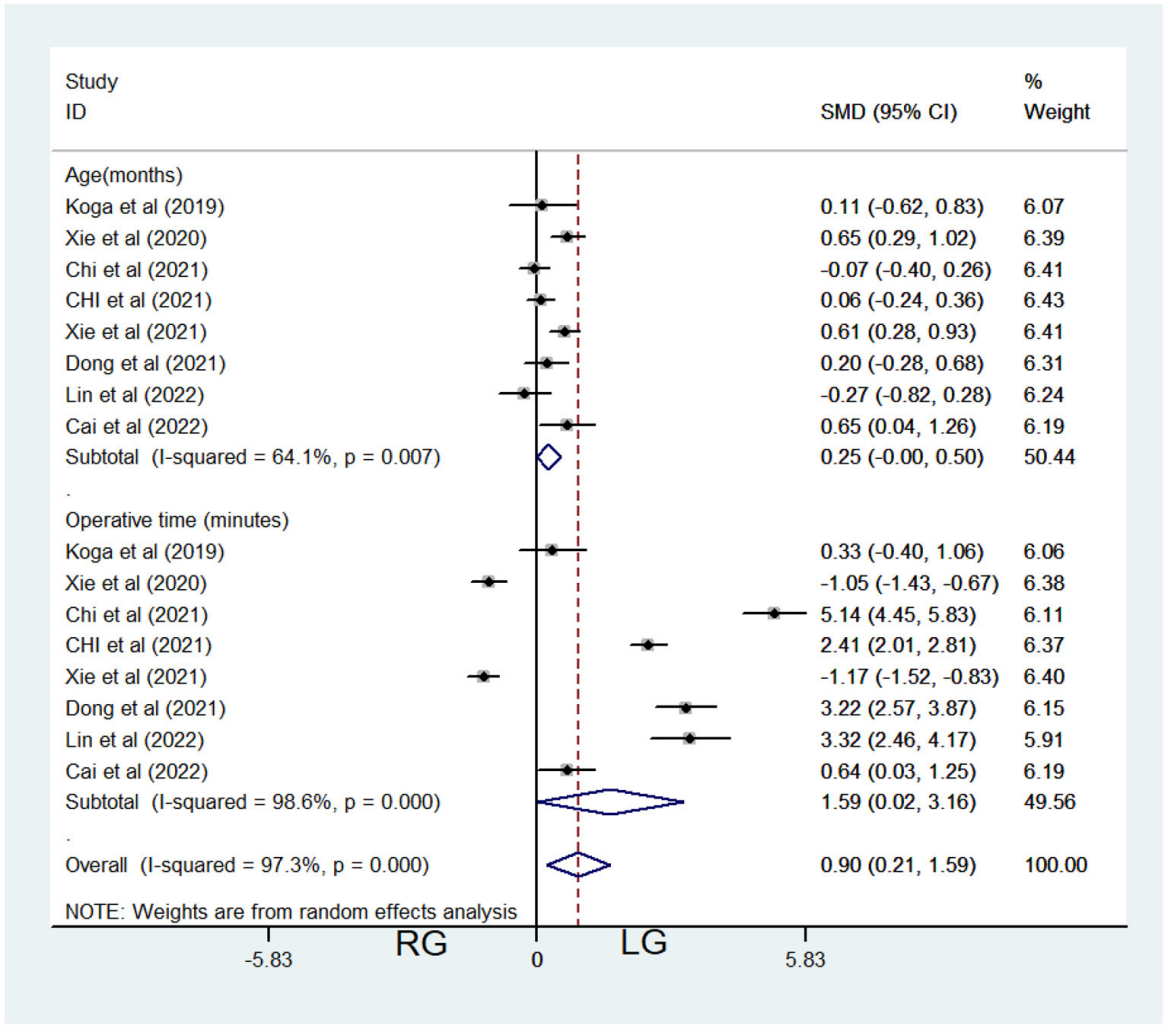
Comparison of age at surgery and operative time between the robotic-assisted operation group (RG) and laparoscopic operation group (LG).

Eight studies contributed data about RG and LG, including 862 patients (532 in the LG and 330 in the RG, Table 1). Random-effects model was applied because of significant heterogeneity (*I*^2^ = 64.1%, *P* < 0.001, Table 3). Meta-analysis showed no significant difference between the 2 groups [standardized mean difference (SMD) = 0.25, 95% CI = (−0.001, 0.50), *P* = 0.051 > 0.05; Figure 1].

**Table 3:** Outcomes of meta-analysis between robotic-assisted operation group (RG) and laparoscopic operation group (LG) robotic-assisted operation group and open operation group (OG).

**Subgroups**	**Number of studies**	**Tests of heterogeneity**		**Effects model**	**Meta-analysis**	
		***P*-Value**	***I^2^ %***		**Effects size (95% CI)**	***P*-Value**
**RG vs. LG**	51					
Age	8	<0.001^*^	64.1	Random	0.25 (−0.001, 0.50)	0.051
Operative time	8	<0.001^*^	98.6	Random	1.59 (0.02, 3.16)	0.047^*^
Blood loss	8	<0.001^*^	97.8	Random	−1.52 (−2.71, −0.32)	0.013^*^
Time to enteral feeding	8	<0.001^*^	84.2	Random	−0.83 (−1.22, −0.44)	<0.001^*^
Hospital stays	8	<0.001^*^	86.7	Random	−0.81 (−1.23, −0.38)	<0.001^*^
Complications^a^	8	0.063	47.7	Fixed	1.09 (1.04, 1.13)	<0.001^*^
Expense	3	<0.001^*^	95.5	Random	8.58 (5.27, 11.89)	<0.001^*^
**RG vs. OG**	18					
Age	3	0.805	0.00	Fixed	0.46 (0.26, 0.66)	<0.001^*^
Operative time	3	<0.001^*^	96.3	Random	3.96 (2.38, 5.55)	<0.001^*^
Blood loss	3	0.019^*^	74.8	Random	−0.16 (−0.75, 0.25)	0.454
Time to enteral feeding	3	<0.001^*^	98.7	Random	−1.70 (−3.77, 0.36)	0.106
Hospital stays	3	<0.001^*^	90.4	Random	−0.93 (−1.62, −0.25)	0.008^*^
Complications^a^	3	0.262	25.3	Fixed	0.97 (0.92, 1.01)	0.147

RG, robotic-assisted operation group; LG, laparoscopic operation group; OG, open operation group.

* P < 0.05 was considered to be statistically significant;

a Complications included short-term and long-term complications (see the paragraph of “Post-Operative complications” for detail).

##### RG vs. OG

Three studies contributed data about RG and OG, including 628 patients (497 in the OG group and 131 in the RG, Table 1). Fixed-effects model was applied because of low heterogeneity (*I*^2^ = 0.00%, *P* = 0.805 > 0.05, Table 3). Meta-analysis showed significant difference between the 2 groups (SMD = 0.46, 95% CI = (0.26, 0.66), *P* < 0.001), which demonstrated significantly older age of RG (**Figure 3**).

#### Operative time

##### RG vs. LG

Eight studies contributed data about RG and LG, including 862 patients (532 in the LG and 330 in the RG, Table 1). Random-effects model was applied because of significant heterogeneity (*I*^2^ = 98.6%, *P* < 0.001, Table 3). Meta-analysis showed significant difference between the 2 groups [SMD = 1.59, 95% CI = (0.02, 3.16), *P* = 0.047 <0.05], which demonstrated significantly longer operative time of RG (Figure 1). In Koga's report (7), the average operation time of the two groups was 618 and 654 min, respectively (including the robot docking time), which were significantly longer than the other seven reports.

##### RG vs. OG

Three studies contributed data about RG and OG, including 628 patients (497 in the OG group and 131 in the RG, Table 1). Random-effects model was applied because of significant heterogeneity (*I*^2^ = 96.3%, *P* < 0.001, Table 3). Meta-analysis showed significant difference between the two groups [SMD = 3.96, 95% CI = (2.38, 5.55), *P* < 0.001], which demonstrated significantly longer operative time of RG (**Figure 3**).

#### Blood loss

##### RG vs. LG

**Figure 2:**
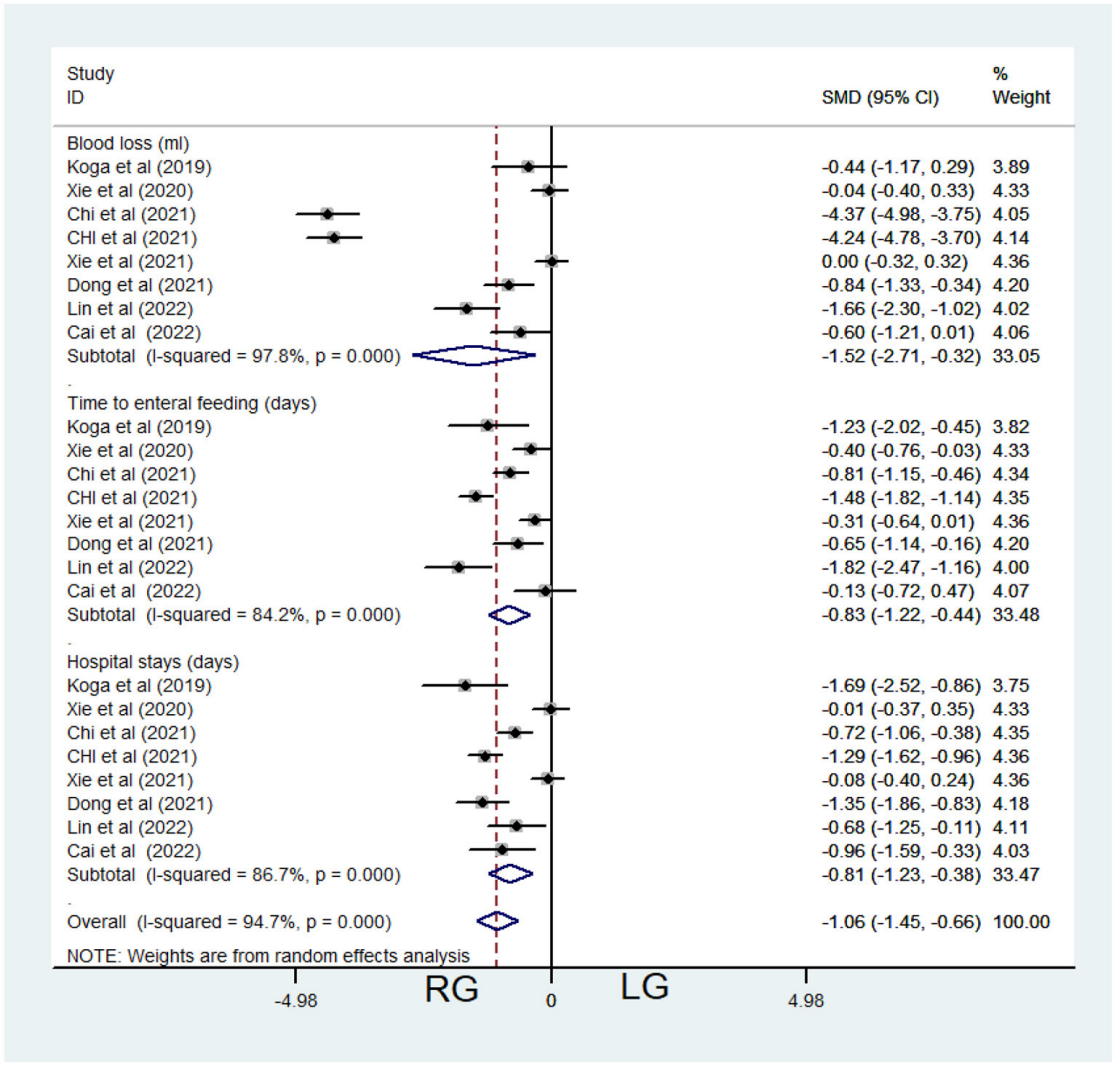
Comparison of post-operative characteristics between the robotic-assisted operation group (RG) and laparoscopic operation group (LG).

Eight studies contributed data about RG and LG, including 862 patients (532 in the LG and 330 in the RG, Table 1). Random-effects model was applied because of significant heterogeneity (*I*^2^ = 97.8%, *P* < 0.001, Table 3). Meta-analysis showed significant difference between the 2 groups [SMD = −1.52, 95% CI = (−2.71, −0.32), *P* = 0.013 <0.05], which demonstrated significantly less blood loss of RG (Figure 2).

##### RG vs. OG

**Figure 3:**
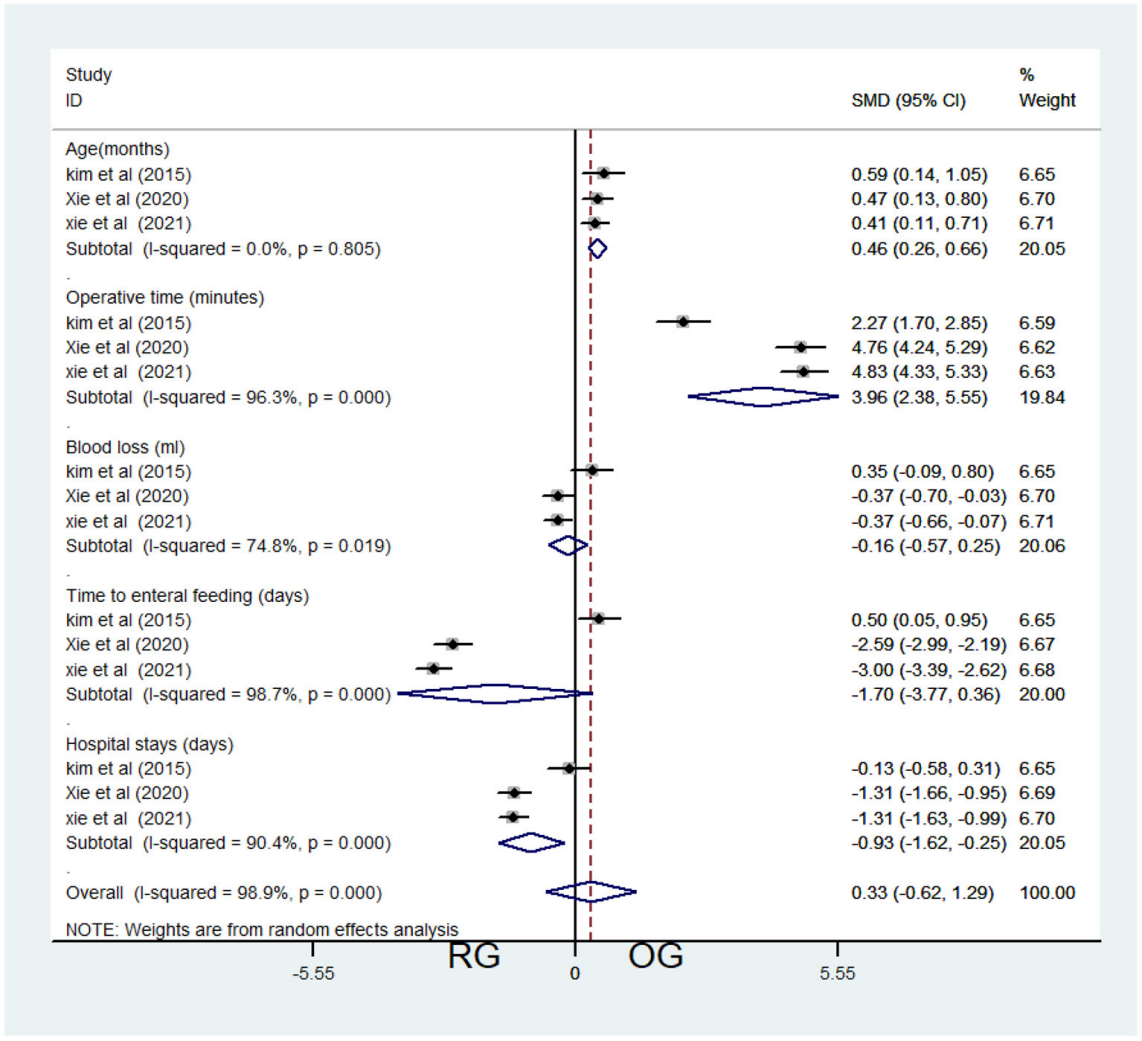
Comparison of baseline and post-operative characteristics between the robotic-assisted operation group (RG) and open operation group (OG).

Three studies contributed data about RG and OG, including 628 patients (497 in the OG group and 131 in the RG, Table 1). Random-effects model was applied because of significant heterogeneity (*I*^2^ = 74.8%, *P* = 0.019 < 0.05, Table 3). Meta-analysis showed no significant difference between the 2 groups [SMD = −0.16, 95% CI = (−0.75, 0.25), *P* = 0.454; Figure 3].

#### Time to enteral feeding

##### RG vs. LG

Eight studies contributed data about RG and LG, including 862 patients (532 in the LG and 330 in the RG, Table 1). Random-effects model was applied because of significant heterogeneity (*I*^2^ = 84.2%, *P* < 0.001, Table 3). Meta-analysis showed significant difference between the 2 groups [SMD = −0.83, 95% CI = (−1.22, −0.44), *P* < 0.001], which demonstrated significantly shorter time to enteral feeding of RG (Figure 2).

##### RG vs. OG

Three studies contributed data about RG and OG, including 628 patients (497 in the OG group and 131 in the RG, Table 1). Random-effects model was applied because of significant heterogeneity (*I*^2^ = 98.7%, *P* < 0.001, Table 3). Meta-analysis showed no significant difference between the two groups [SMD = −1.70, 95% CI = (−3.77, 0.36), P=0.106; Figure 3].

#### Hospital stays

##### RG vs. LG

Eight studies contributed data about RG and LG, including 862 patients (532 in the LG and 330 in the RG, Table 1). Random-effects model was applied because of significant heterogeneity (*I*^2^ = 86.7%, *P* < 0.001, Table 3). Meta-analysis showed significant difference between the 2 groups [SMD = −0.81, 95% CI = (−1.23, −0.38), *P* < 0.001], which demonstrated significantly shorter time to stay in the hospital in RG (Figure 2).

##### RG vs. OG

Three studies contributed data about RG and OG, including 628 patients (497 in the OG group and 131 in the RG, Table 1). Random-effects model was applied because of significant heterogeneity (*I*^2^ = 90.4%, *P* < 0.001, Table 3). Meta-analysis showed no significant difference between the two groups [SMD = −0.93, 95% CI = (−1.62, −0.25), *P* = 0.008 < 0.05; Figure 3].

#### Post-operative complications

We assessed all the complications reported by the enrolled articles, including long-term complications, short-term complications, and total post-operative complications. Short-term complications included bile leakage (most common), bleeding, intestinal obstruction, wound infection, acute pancreatitis, fluid collection, vein thrombus, etc. Long-term complications included anastomotic strictures (most common), cholelithiasis, residual cysts, etc. Overall, we take total post-operative complications into consideration in this meta-analysis.

##### RG vs. LG

**Figure 4:**
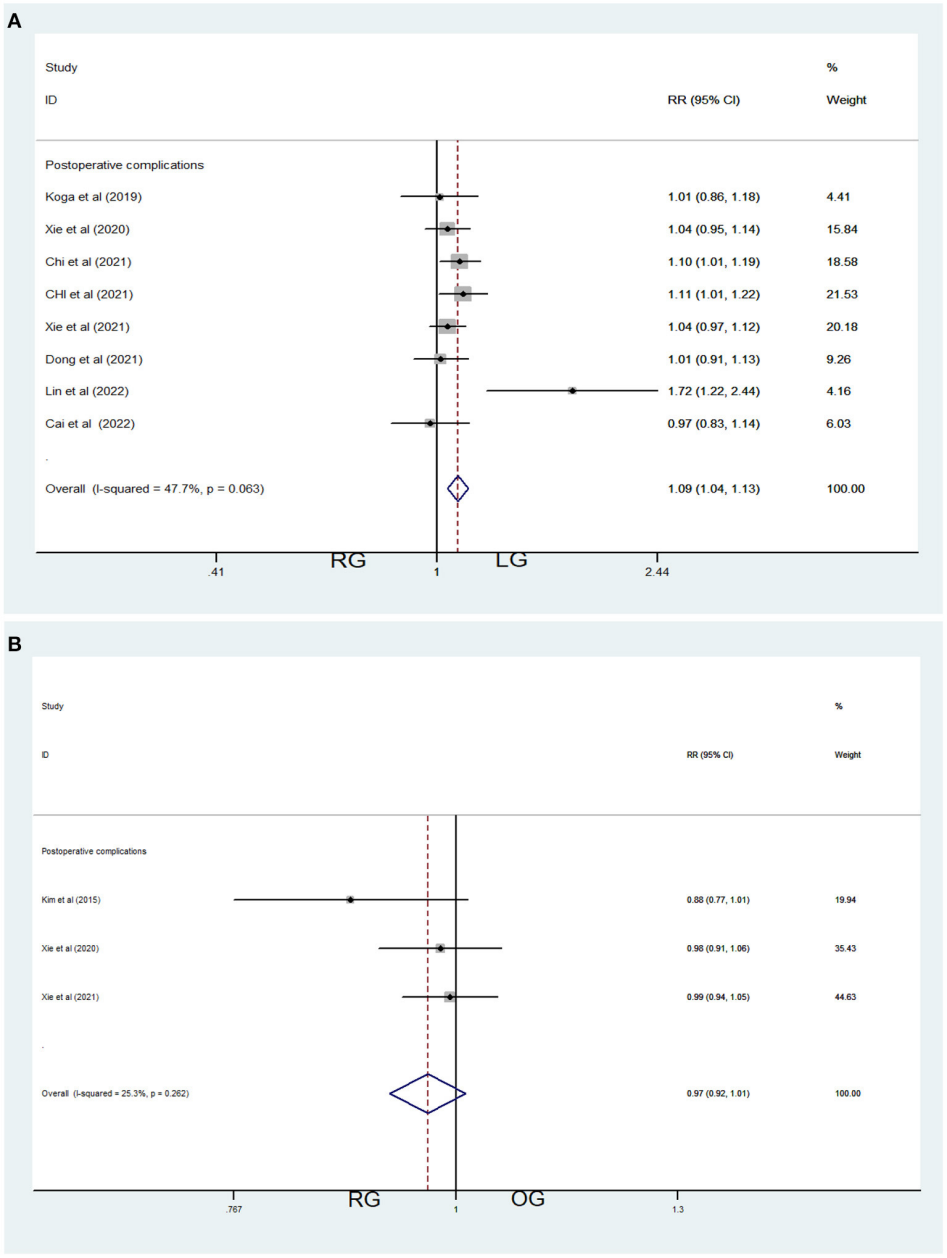
Comparations of post-operative complications in robotic-assisted operation group (RG), laparoscopic operation group (LG) and open operation group (OG). **(A)** Post-operative complications of RG vs. LG **(B)** Post-operative complications of RG vs. OG.

Eight studies contributed data about RG and LG, including 862 patients (532 in the LG and 330 in the RG, Table 1). Fixed-effects model was applied because of low heterogeneity (*I*^2^ = 47.7%, *P* = 0.063 > 0.05, Table 3). Meta-analysis showed significant difference between the 2 groups [Relative risk (RR) =1.09, 95% CI = (1.04, 1.13), *P* < 0.001], which demonstrated significantly fewer post-operative complications of RG (Figure 4A).

##### RG vs. OG

Three studies contributed data about RG and OG, including 628 patients (497 in the OG group and 131 in the RG, Table 1). Fixed-effects model was applied because of low heterogeneity (*I*^2^ = 25.3%, *P* = 0.262 > 0.05, Table 3). Meta-analysis showed no significant difference between the two groups [RR = 0.97, 95% CI = (0.92, 1.01), *P* = 0.147] (Figure 4B).

#### Expense

**Figure 5:**
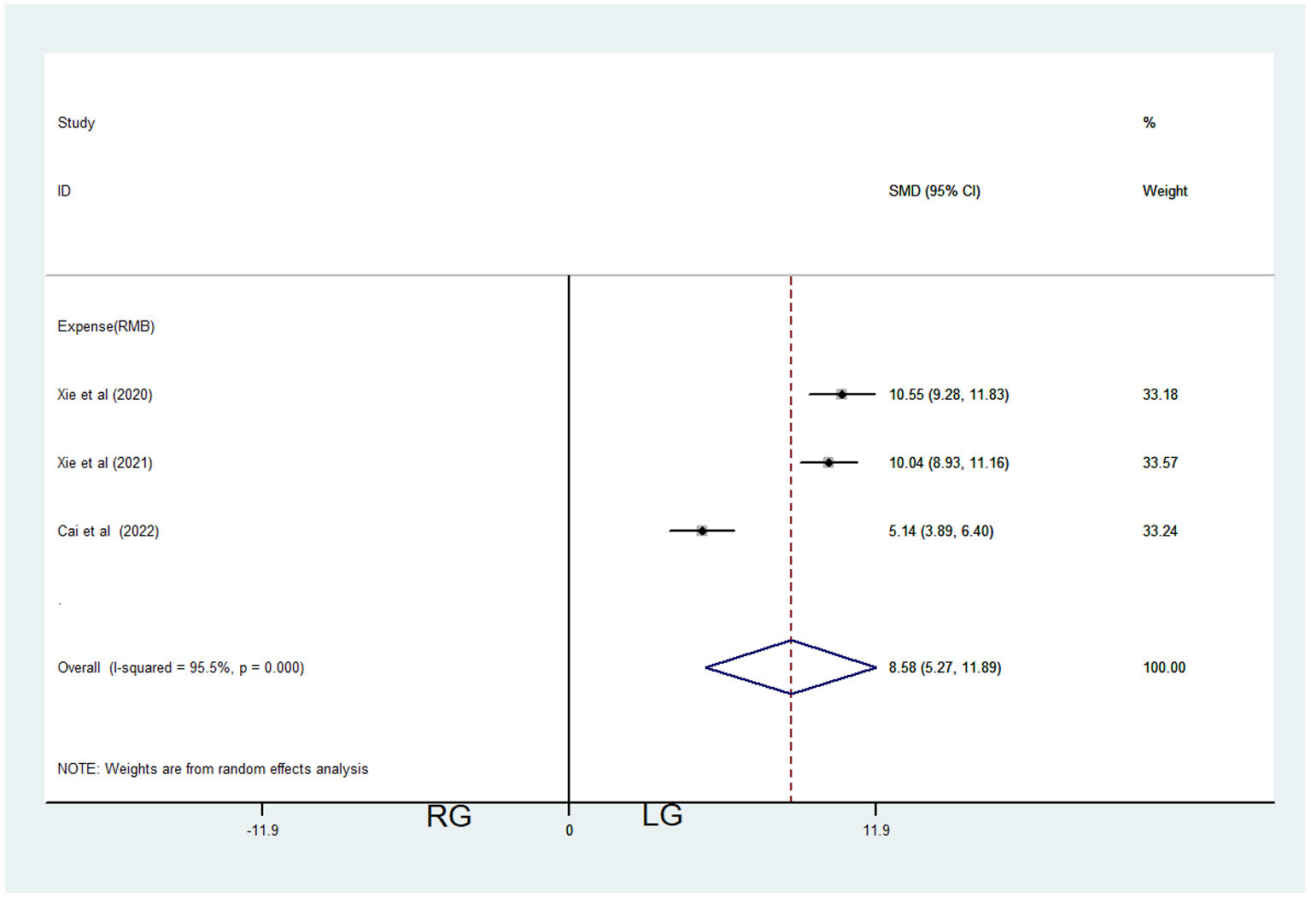
Comparations of expenses in robotic-assisted operation group (RG) and laparoscopic operation group (LG).

Only three studies contributed data about RG and LG, including 361 patients (241 in the LG and 120 in the RG, Table 1). Random-effects model was applied because of significant heterogeneity (*I*^2^ = 95.5%, *P* < 0.001, Table 3). Meta-analysis showed significant difference between the 2 groups [SMD = 8.58, 95% CI = (5.27, 11.89), *P* < 0.001], which demonstrated significantly higher expenses of RG (Figure 5) (1¥ = 0.149$).

### Publication bias

**Figure 6:**
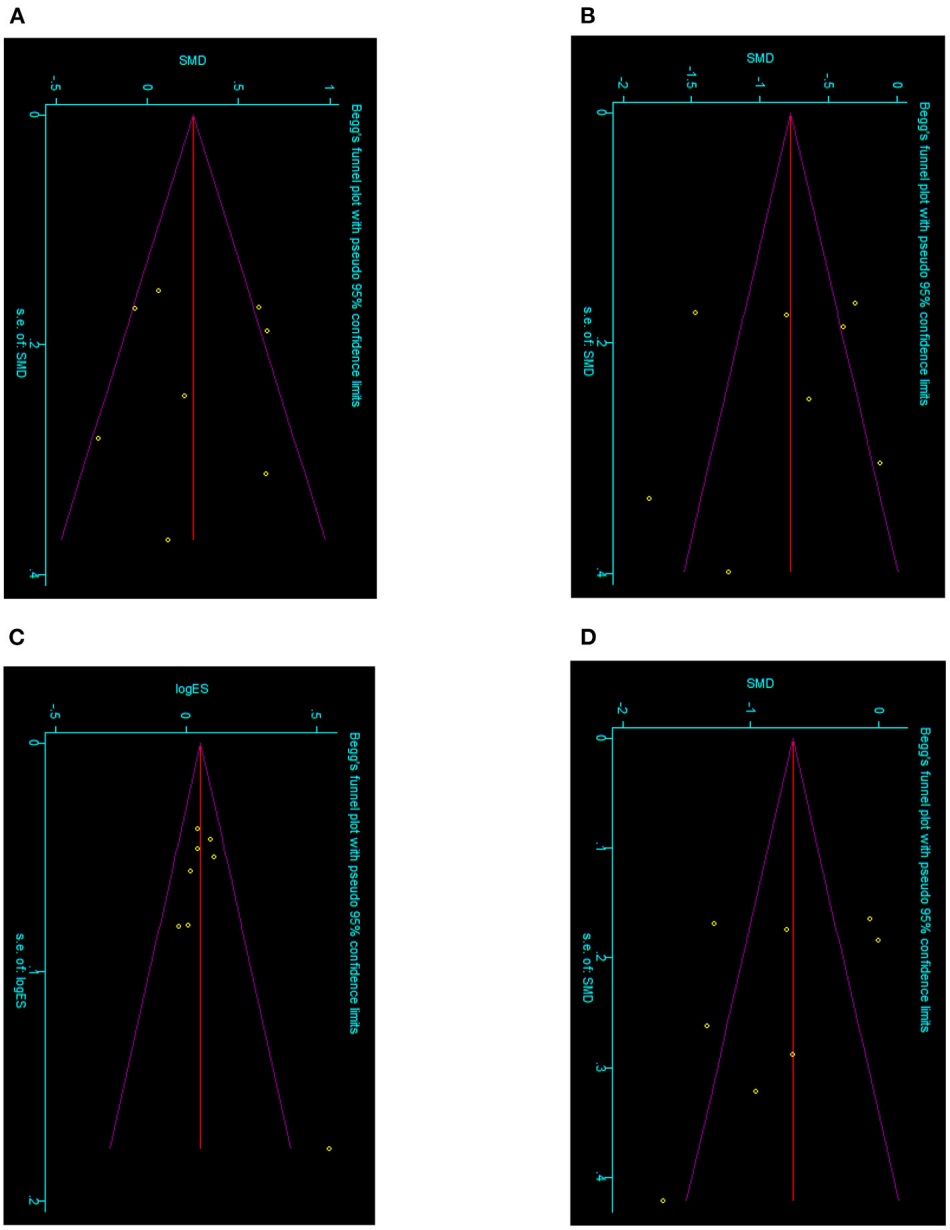
Begg's funnel plots of classified subgroups of the enrolled records. **(A)** Meta-analysis of age between RG and LG. **(B)** Meta-analysis of time to enteral feeding between RG and LG. **(C)** Meta-analysis of post-operative complications between RG and LG. **(D)** Meta-analysis of hospital stays between RG and LG.

Begg's Test and Egger's Test were conducted and Begg's funnel plots was drawn for the enrolled nine records. Different subgroups were classified to evaluate the publication bias (Table 4). Some basically symmetrical inverted funnels were exhibited (Figures 6A–D), and the publications with significant publication bias were excluded.

**Table 4:** Begg's and Egger's Test of publication bias of robotic-assisted operation group (RG) and laparoscopic operation group (LG).

**Subgroups**	**Number of studies**	**Begg's test/Egger's test**
		*P*-Value^*^
**RG vs. LG**	51	
Age	8	0.902/0.941
Operative time	8	0.266/0.091
Blood loss	8	0.386/0.138
Time to enteral feeding	8	0.536/0.631
Hospital stays	8	0.536/0.282
Post-operative complications	8	0.902/0.345

* P value means the value of Pr > |z| (continuity corrected, in Begg's Test) or P > |t| (in Egger's Test). P > 0.05 was considered to have a low risk of publication bias.

## Discussion

For decades, numerous studies had demonstrated the safety and efficacy of laparoscopic surgery for choledochal cysts (CC). Nowadays, robotic-assisted technology has become an important part of minimally invasive surgery with nearly two decades of development. The emergence of robot-assisted operation relieved the fatigue of operators and shortened the learning curve of endoscopic surgery: the learning curve for robotic-assisted cyst excision and Roux-en-Y hepaticojejunostomy was only 14 cases in pediatric patients (17). Meehan and Sandler (18) reported that a baby weighted 2.2 kg was treated by robotic-assisted abdominal surgery in 2007, and pointed out that the advantages of robotic-assisted surgery over laparoscopic operation. In the same year, Meehan et al. (19) reported his experience in hepatobiliary surgery in children, and pointed out that robotic-assisted surgery was safe and effective for choledochal cyst and biliary atresia. Robot-assisted thoracic surgery, including mediastinal tumor and lobectomy, had also been reported (20, 21). Robot-assisted surgery has been able to complete almost all pediatric endoscopic surgery nowadays. However, the obstruction of its application in the field of pediatric abdominal surgery is that small abdominal cavity and limited operating space of the child are difficult to match the huge size of the machine. Koga et al. argued that robotic surgical system (RSS) was not suitable of dissecting tissue in children because of space constraints and a limited range of energy devices (7). Kim et al. reported on the technical limitations of robot-assisted CC resection in children: They excluded patients with severely inflamed, fragile peribiliary tissue of the bile duct from robotic surgery due to difficulties with hemostasis, and also excluded patients with protein plugs or obstructed stones which are difficult to remove in the distal common channel while using RSS. They eventually converted to open surgery to remove impacted calculi in the distal common channel (9). Nevertheless, there are no meta-analysis comparing post-operative outcomes of traditional open, laparoscopic and robotic-assisted CC resection and Roux-en-Y hepaticojejunostomy in children. The purpose of our meta-analysis was to compare outcomes and reliabilities among the 3 approaches in total.

Overall, we enrolled 9 retrospective cohort studies, included 1,395 children. Compared to laparoscopic operation group (LG), subgroup analysis demonstrated the robotic-assisted operation group (RG) had significant longer operative time [SMD = 1.59, 95% CI = (0.02, 3.16), *P* < 0.05], less blood loss [SMD = −1.52, 95% CI = (−2.71, −0.32), *P* < 0.05], shorter enteral feeding time [SMD = −0.83, 95% CI = (−1.22, −0.44), *P* < 0.001], shorter time to stay in the hospital [SMD = −0.81, 95% CI = (−1.23, −0.38), *P* < 0.001], fewer post-operative complications [RR =1.09, 95% CI = (1.04, 1.13), *P* < 0.001] and higher expenses [SMD = 8.58, 95% CI = (5.27, 11.89), *P* < 0.001], while the age at surgery seemed to have no significant differences [SMD = 0.25, 95% CI = (−0.001, 0.50), *P* = 0.051 > 0.05], but there was expressly tendency to a younger age in LG for *P* = 0.051 > 0.05. Due to the development of surgical instruments and surgical experiences, laparoscopic surgery has occupied a dominant position for decades. Without the restriction of age or weight, laparoscopy could be applied in more complicated CC children (22, 23). Laparoscopy allowed more flexibility of manipulating instruments and positions, which were limited in RG once the dock was complete, and the operative time of laparoscopic operations was reduced as a result. The robotic technique overcomes many of the limitations which were encountered in laparoscopic surgery, including a greater range of motion for instruments and 3D high-definition vision for better depth perception. The functions of the robotic arms most related to precise meticulous surgery that RSS has a reputation for are tremor filtering and motion scaling, which converted large movements of operator to tiny movements of instrument, improving the operator's dexterity greatly (24). Suturing during an anastomosis and knot tying by robotic arms are much easier than unarticulated laparoscopic instruments in a small peritoneal cavity, because the operator can sit comfortably in front of the console (7). These maybe the reasons why RG had those better outcomes than LG. Besides, total expenses were much higher in the RG than in the LG, which increased the economic burden on patients. The higher surgical costs would influence the choice of operational management (24).

With respect to open operation group (OG), subgroup analysis demonstrated the robotic-assisted operation group (RG) had significant older age [SMD = 0.46, 95% CI = (0.26, 0.66), *P* < 0.001], longer operative time [SMD = 3.96, 95% CI = (2.38, 5.55), *P* < 0.001] and shorter time to stay in the hospital [SMD = −0.93, 95% CI = (−1.62, −0.25), *P* < 0.05], while the blood loss [SMD = −0.16, 95% CI = (−0.75, 0.25), *P* > 0.05], time to enteral feeding [SMD = −1.70, 95% CI = (−3.77, 0.36), *P* > 0.05] and post-operative complications [RR =0.97, 95% CI = (0.92, 1.01), *P* > 0.05] seemed to have no significant differences. Lacking of force feedback was one of the concerns for robotic-assisted operation which would further affect the precise anatomy of adjacent organs or adhesive tissues, especially in those less-experienced hands. Open operation was the most traditional and earliest procedure for CC (1), this strategy was always stable and still popular in some area for many reasons: it was a more experienced technique and required less effort to connect the pediatric hepatic duct to the intestine. In the early time, only the complicated CC or the redo-hepaticojejunostomy children would choose open operation, so that the operator could get precise and intuitionistic feedback at the same time. A report in 2010 of the United States showed that incidence of conversion to open surgery was 4% in a robotic-assisted group of children with an average surgical age of 8.6 years (25). Because of no technical limitations of operational instruments, it would be a shorter time and a wider surgical visual field to complete the surgery in OG, the age at surgery would be younger and hemostasis under direct vision would more enough. However, several wide and terrible wounds would be left, which resulted in a longer time to stay in hospital and some short-term complications, such as wound infection and bleeding (But in our meta-analysis, there was no significant difference in post-operative complications). Minimal invasive surgery in children has been widely practiced for a variety of conditions, there has been an increasing number of parents asking about safe minimally invasive operations for complex procedures for their children (9). A multi-center clinical study in France showed that there was no difference in the effect of robotic surgery between children weighed above and below 15.0 kg: “size does not affect surgery success,” but operators needed to adjust the patient's posture and the position of abdominal puncture device carefully (26). The overall height during the operation, patient's proper position, puncture device position, and appearance of smaller instruments could promote the safety of the children's operation, avoiding crushing injury. Keeping the mobility of mechanical arms is the premise to complete operation smoothly and efficiently (27).

There are some limitations in our meta-analysis. First, the included literatures were all retrospective cohort studies, lacking randomized controlled studies, and thus selection bias was inevitable. Second, the surgical teams were also the report authors, and there might be a certain risk of bias. Third, only two records were analyzed and some post-operative outcomes were heterogeneous significantly. In addition, further long-term follow-up is required.

In summary, laparoscopic and robotic-assisted procedure are both safe and minimal invasive operational strategies, which have its own specialty (24). Robotic-assisted procedure was latter and developed on the basis of laparoscopy, which may slowly surpass and has a trend to replace laparoscopy for its advantages. Certainly, the arms of the robot would be infinitely approaching the arms of the operator but not real, more experiences should be accumulated for the unexpected complexities, so as to be more stable in the younger age of children.

## Conclusion

Laparoscopic and robotic-assisted procedure are both safe and minimal invasive operational strategies. Robotic-assisted procedure may slowly surpass and has a trend to replace laparoscopy for its advantages. More experiences in robotic-assisted operation should be accumulated for the unexpected complexities, so as to be more stable in the younger age of children.

## Data availability statement

The original contributions presented in the study are included in the article/supplementary material, further inquiries can be directed to the corresponding author/s.

## Author contributions

SX and YHu: systematic search, data extraction, formal analysis, and article writing. YHe: formal analysis and quality assessment. ML and DW: quality assessment. YF: determined the main idea of the manuscript. All authors reviewed the manuscript. All authors contributed to the article and approved the submitted version.

## Conflict of interest

The authors declare that the research was conducted in the absence of any commercial or financial relationships that could be construed as a potential conflict of interest.

## Publisher's note

All claims expressed in this article are solely those of the authors and do not necessarily represent those of their affiliated organizations, or those of the publisher, the editors and the reviewers. Any product that may be evaluated in this article, or claim that may be made by its manufacturer, is not guaranteed or endorsed by the publisher.

## References

[1] Ishibashi H Shimada M Kamisawa T Fujii H Hamada Y Kubota M . Japanese clinical practice guidelines for congenital biliary dilatation. J Hepatobiliary Pancreat Sci. (2017) 24:1–16. 10.1002/jhbp.415 28111910

[2] Farello GA Cerofolini A Rebonato M Bergamaschi G Ferrari C Chiappetta A. Congenital choledochal cyst: video-guided laparoscopic treatment. Surg Laparosc Endosc. (1995) 5:354–8. 8845978

[3] Woo R Le D Albanese CT Kim SS. Robot-assisted laparoscopic resection of a type I choledochal cyst in a child. J Laparoendosc Adv Surg Tech A. (2006) 16:179–83. 10.1089/lap.2006.16.179 16646713

[4] Chi SQ Cao GQ Li S Guo JL Zhang X Zhou Y . Outcomes in robotic versus laparoscopic-assisted choledochal cyst excision and hepaticojejunostomy in children. Surg Endosc. (2021) 35:5009–14. 10.1007/s00464-020-07981-y 32968912

[5] Lin S Chen J Tang K He Y Xu X Xu D. Trans-umbilical single-site plus one robotic assisted surgery for choledochal cyst in children, a comparing to laparoscope-assisted procedure. Front Pediatr. (2022) 10:806919. 10.3389/fped.2022.806919 35281244 PMC8914220

[6] Chi SQ Xu YH Tang ST Zhang X Cao GQ Li S. Comparison between robot-assisted and traditional laparoscopic surgery on choledochal cyst excision and hepaticojejunostomy in children. Chin J Robot Surg. (2021) 2:248–254. 10.12180/j.issn.2096-7721.2021.04.002

[7] Koga H Murakami H Ochi T Miyano G Lane GJ Yamataka A. Comparison of robotic versus laparoscopic hepaticojejunostomy for choledochal cyst in children: a first report. Pediatr Surg Int. (2019) 35:1421–5. 10.1007/s00383-019-04565-3 31555861

[8] Xie X Li K Wang J Wang C Xiang B. Comparison of pediatric choledochal cyst excisions with open procedures, laparoscopic procedures and robot-assisted procedures: a retrospective study. Surg Endosc. (2020) 34:3223–31. 10.1007/s00464-020-07560-1 32347390

[9] Kim NY Chang EY Hong YJ Park S Kim HY Bai SJ . Retrospective assessment of the validity of robotic surgery in comparison to open surgery for pediatric choledochal cyst. Yonsei Med J. (2015) 56:737–43. 10.3349/ymj.2015.56.3.737 25837180 PMC4397444

[10] Xie XL Li KW Wang C Wang Q Li F Xiang B. Clinical efficacy of Da Vinci (SI) robot-assisted choledochal cyst excision in pediatrics. Chin J Pediatr Surg. (2021) 42:610–6. 10.1016/j.jpedsurg.2020.07.019 32829883

[11] Dong L Chu Z Cui X Zhang D Wang J. A comparative study of Da Vinci robot versus traditional laparoscopy for congenital choledochal cyst in children. Chin J Pediatr Surg. (2021) 42:17–22. 10.3760/cma.j.cn421158-20191022-00603

[12] Cai D Chen Q Zhang L Zhang Y Pan T Chen K . Comparative study of Da Vinci technique versus traditional laparoscopic technique in the treatment of choledochal cyst. J Clin Pediatr Surg. (2022) 21:51–57. 10.3760/cma.j.cn.101785-202012066-010

[13] Moher D Liberati A Tetzlaff J Altman DG PRISMA Group. Preferred reporting items for systematic reviews and meta-analyses: the PRISMA statement. Ann Intern Med. (2009) 151:264–9. 10.7326/0003-4819-151-4-200908180-00135 19622511

[14] Stang A. Critical evaluation of the Newcastle-Ottawa scale for the assessment of the quality of nonrandomized studies in meta-analyses. Eur J Epidemiol. (2010) 25:603–5. 10.1007/s10654-010-9491-z 20652370

[15] Luo D Wan X Liu J Tong T. Optimally estimating the sample mean from the sample size, median, mid-range, and/or mid-quartile range. Stat Methods Med Res. (2018) 27:1785–805. 10.1177/0962280216669183 27683581

[16] Wan X Wang W Liu J Tong T. Estimating the sample mean and standard deviation from the sample size, median, range and/or interquartile range. BMC Med Res Methodol. (2014) 14:135. 10.1186/1471-2288-14-135 25524443 PMC4383202

[17] Xie X Feng L Li K Wang C Xiang B. Learning curve of robot-assisted choledochal cyst excision in pediatrics: report of 60 cases. Surg Endosc. (2021) 35:2690–7. 10.1007/s00464-020-07695-1 32556766

[18] Meehan JJ Sandler A. Robotic repair of a Bochdalek congenital diaphragmatic hernia in a small neonate: robotic advantages and limitations. J Pediatr Surg. (2007) 42:1757–60. 10.1016/j.jpedsurg.2007.06.013 17923210

[19] Meehan JJ Elliott S Sandler A. The robotic approach to complex hepatobiliary anomalies in children: preliminary report. J Pediatr Surg. (2007) 42:2110–4. 10.1016/j.jpedsurg.2007.08.040 18082719

[20] Meehan JJ Sandler AD. Robotic resection of mediastinal masses in children. J Laparoendosc Adv Surg Tech A. (2008) 18:114–9. 10.1089/lap.2007.0092 18266588

[21] Meehan JJ Phearman L Sandler A. Robotic pulmonary resections in children: series report and introduction of a new robotic instrument. J Laparoendosc Adv Surg Tech A. (2008) 18:293–95. 10.1089/lap.2007.0078 18373461

[22] Diao M Li L Cheng W. Timing of surgery for prenatally diagnosed asymptomatic choledochal cysts: a prospective randomized study. J Pediatr Surg. (2012) 47:506–12. 10.1016/j.jpedsurg.2011.09.056 22424346

[23] Ngoc Son T Thanh Liem N Manh Hoan V. One-staged or two-staged surgery for perforated choledochal cyst with bile peritonitis in children? A single center experience with 27 cases. Pediatr Surg Int. (2014) 30:287–90. 10.1007/s00383-014-3461-6 24463980

[24] Yin T Chen S Li Q Huang T Li L Diao M. Comparison of outcomes and safety of laparoscopic and robotic-assisted cyst excision and hepaticojejunostomy for choledochal cysts: a systematic review and meta-analysis. Ann Med Surg. (2022) 75:103412. 10.1016/j.amsu.2022.103412 35386800 PMC8977927

[25] Sorensen MD Johnson MH Delostrinos C Bice JB Grady RW Lendvay TS. Initiation of a pediatric robotic surgery program: institutional challenges and realistic outcomes. Surg Endosc. (2010) 24:2803–8. 10.1007/s00464-010-1052-8 20376494

[26] Ballouhey Q Villemagne T Cros J Szwarc C Braik K Longis B . A comparison of robotic surgery in children weighing above and below 15.0 kg: size does not affect surgery success. Surg Endosc. (2015) 29:2643–50. 10.1007/s00464-014-3982-z 25480612

[27] Chang C Steinberg Z Shah A Gundeti MS. Patient positioning and port placement for robot-assisted surgery. J Endourol. (2014) 28:631–8. 10.1089/end.2013.0733 24548088 PMC4382714

